# Manipulation on radiation angles via spatially organized multipoles with vertical split-ring resonators

**DOI:** 10.1515/nanoph-2023-0386

**Published:** 2023-10-05

**Authors:** Hao-Yuan Tsai, Che-Chin Chen, Chun-Yen Chen, Yi-Jie Lin, Wei-Chun Chen, Hung-Pin Chen, Yu-Wei Lin, Takuo Tanaka, Ta-Jen Yen

**Affiliations:** Materials Science and Engineering, National Tsing Hua University, Hsinchu, Taiwan; Taiwan Instrument Research Institute, National Applied Research Laboratories, Hsinchu, 30076, Taiwan; Metamaterials Laboratory, RIKEN Cluster for Pioneering Research, 2-1 Hirosawa, Wako, Saitama, 351-0198, Japan; Innovative Photon Manipulation Research Team, RIKEN Center for Advanced Photonics, 2-1 Hirosawa, Wako, Saitama, 351-0198, JAPAN

**Keywords:** angular reconfiguration, metamaterials, split ring resonator, three-dimensional metamaterials, infrared radiation, nanoantenna

## Abstract

Herein, the radiation patterns of single-split ring resonators (SSRRs) and double-split ring resonators (DSRRs) in the vertical direction are tailored by reconfiguring the resonator geometries. To design unequal arm lengths for controlling the floating split angle of the resonators and changing their electromagnetic multipole compositions, vertical metamaterials were fabricated using the metal-stress-driven self-folding method. The simulation results well agree with the experimental transmittance and reflectance results and demonstrate the geometry-dependent angle variation of the far-field radiation. Symmetric SSRRs and DSRRs radiate in the vertical and horizontal directions, respectively. With increasing pad shift, the radiation angle of the asymmetric SSRR completely rotates toward the horizontal direction along the ring plane, but the DSRRs can rotate only from 0° to 45° to the horizontal plane. Furthermore, by decomposing the multipoles into their constituents, we show that the directional scattering performance can be verified by manipulating the horizontal and vertical components of the electric dipoles. This novel combination of SSRRs and DSRRs can effectively and efficiently reconfigure the radiation direction in the infrared (IR) region, paving the way for color routers, metasurfaces, and directive IR emitters in compact optical metadevices.

## 1 Introduction

The emergence of metamaterials has led to a new paradigm in electromagnetics, enabling the control of light–matter interactions by various approaches. Subwavelength meta-atoms have broadened the definition of materials and enabled high-frequency artificial magnetism [1] and negative refraction [2]. In addition, the intensity [3], phase, and chirality [4] of materials can be controlled by spatially arranging meta-atoms to produce multipolar interferences [5]. Aided by dielectric materials, meta-atoms can preserve both magnetic and electric responses in the optical region. They also demonstrate toroidal dipoles and anapoles [6, 7] having significantly different spatial and temporal symmetry properties. The modulation of novel electromagnetic properties with metamaterials has attracted considerable attention, notably in light detection and ranging [8], optical communication [9, 10], and quantum computation [11]. Specific examples include radiation-directivity modulation with phase-change materials [12], quantum-state modulation with high-frequency artificial magnetism metamaterials obtained from superconductivity materials [11], and focusing-plane modulation by metasurfaces [13].

Most of the reported metamaterials have planar structures on dielectric substrates [14]. Planar metamaterials are strongly influenced by the substrate effect [15]. A dielectric substrate strongly confines the electric and magnetic fields, causing redshift of the resonance. When exposed to the electric and magnetic fields of normal incident light, planar structures are affected only by the former, limiting the response between normal incident light and metamaterials. Consequently, in-plane electric dipoles can only be generated horizontally on a surface [5]. This limitation has challenged the design of nanoantennas. For example, the configuration of a typical nanoantenna mounted on a surface limits the directivity reconfiguration to the horizontal direction [16]. In contrast, a phase-array antenna can be phase-shifted to achieve vertical modulation [17]. However, a compact setup for controlling the phase of each array antenna at high frequency is not yet available, particularly for devices operating above the infrared (IR) region. Geometrically vertical antennas (e.g., three-dimensional (3D) Yagi–Uda antennas [18], vertical cylinders [19], and nanocups structures [20]) that can reconfigure the radiation in the vertical direction have also been reported. Such vertically structured antennas improve the scattering efficiency of normal incident light in different vertical directions from that of planar nanoantennas [21, 22]. However, the complex fabrication processes, uniformity, and lack of angular controllability have hindered the mass production and wide use of these antennas. To resolve these issues, several state-of-art techniques are developed to fabricate 3D-nano structures, such as multi-lithography [23], focused ion beam lithography [24], and 3D-nanoprinting [25]. Chen et al. proposed the metal-stress-driven self-folding method [26], which fabricates vertical structures with high efficiency at low cost. This one-step method accomplishes lithography, deposition, and etching, and can now be implemented in numerous semiconductor factories.

Here, we present two vertical split-ring resonators, namely, single-split ring resonators (SSRRs) and double-split ring resonators (DSRRs). The geometric symmetry of the proposed resonators is broken by introducing uneven arm lengths. By virtue of the vertical geometries, the vertical radiation orientations are controllable over the ranges 0°–180° (180°–360°) for SSRRs and 0°–45° (180°–225°) and 135°–180° (315°–360°) for DSRRs. To demonstrate an angular reconfigurable nanoantenna, we manipulate multipole interferences by varying the magnitudes, directions, and fractions of the electric dipole (ED), magnetic dipole (MD), and electric quadrupole (EQ). The proposed vertical metamaterials provide outstanding capabilities and are promising for numerous mid-IR region applications (such as color routers, metasurfaces, and directive IR emitters in compact optical metadevices).

## 2 Results and discussion

### 2.1 Design of the angular reconfigurable vertical metamaterials

**Figure 1:**
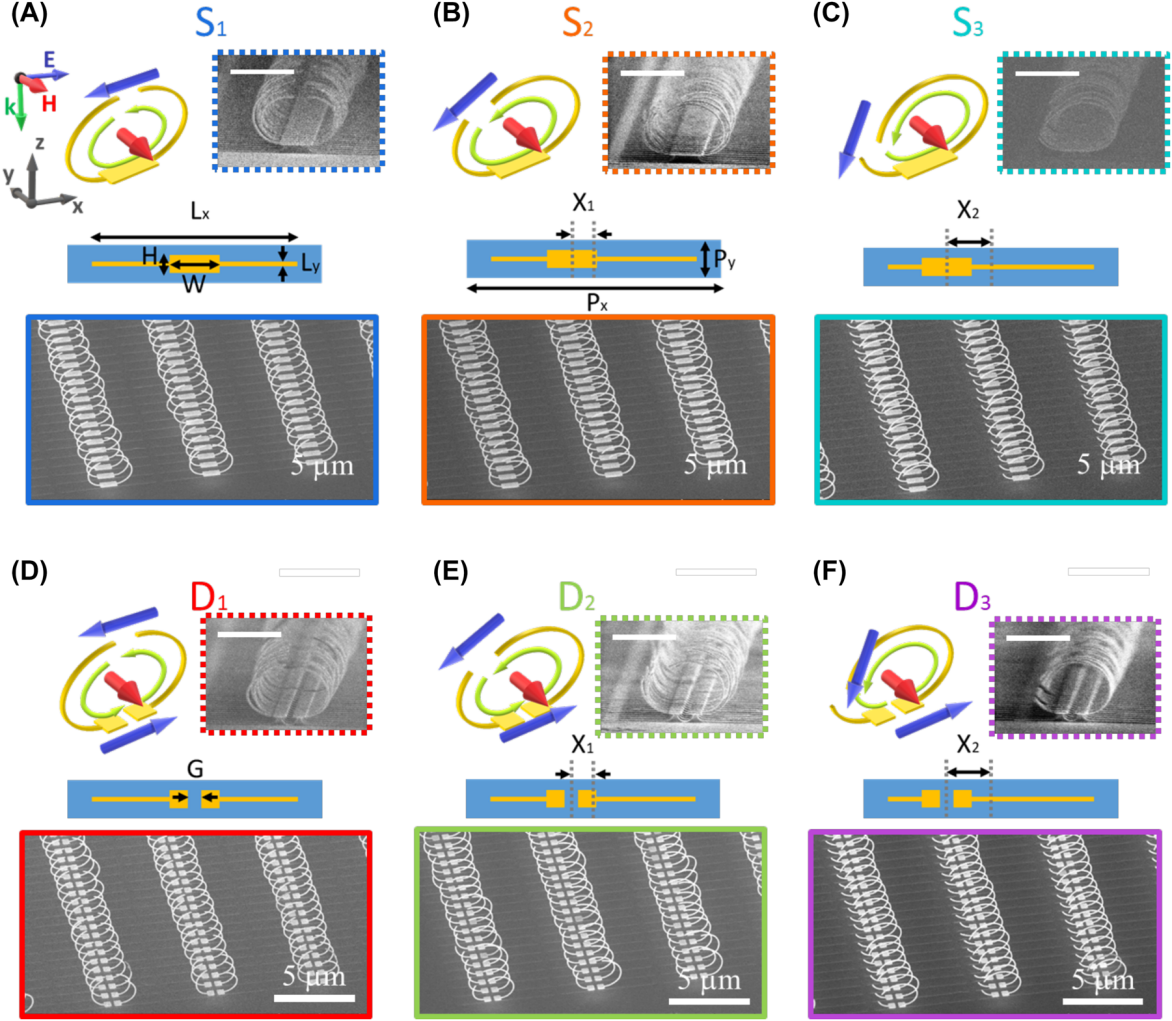
The 3D schematics of the multipoles and induced current configurations (top left), 2D patterns design (center), and fabricated vertical structures (top-right, bottom) of the designed (A–C) SSRRs and (D–F) DSRRs with 0, 0.8, and 1.6 µm pad shifts, denoted as S1(D1), S2(D2), and S3(D3), respectively. Green, red, and blue arrows indicate the induced circular currents, magnetic dipoles, and electric dipoles, respectively. *P*_*x*_, *P*_*y*_, *H*, *W*, and *G* indicated the periodicity in *x* direction, *y* direction, height and width of the pad, and the gap between the pads. *X* is the pad shift distance from the center. Scanning electron microscopy (SEM) images were obtained at 60°-(scale bar, 5 µm) and 85°-(inset, scale bar, 1 µm) stage tilted.

Figure 1 schematizes the vertical metamaterials designed for reconfigurable far-field radiation. To control the elevation angle of far-field radiation patterns, the intensity and direction of three overlapping multipoles (ED, MD, and EQ) in strong electromagnetic bianisotropic vertical SSRRs (Figure 1A) and DSRRs (Figure 1D) are tailored by changing the resonator geometries. Notably, when the designed SSRRs interact with normal incident light polarized along the split direction (*x-*axis in Figure 1A), the ED and MD are generated, as shown in the blue and red arrows in the top-left inset of Figure 1A. However, the EDs at the top and bottom of the DSRRs (Figure 1D) lie in opposite directions and offset each other, allowing the MD and EQ to dominate the resonance. After breaking the symmetry of the vertical SSRRs and DSRRs by introducing unequal arm lengths, the resonators can induce a circular current (green arrow in Figure 1) and excite the ED, which rotates along the split direction due to the resonators’ bianisotropic property.

The vertical SSRRs and DSRRs were efficiently fabricated using the metal-stress-driven self-folding method. This self-assembly process starts from the 2D pattern designs, which are composed of binding-on-substrate pads and two arms, as shown in the center inset of Figure 1A. To facilitate comparisons between the symmetric/asymmetric, and single/double split ring resonators, the pattern length, arm width, periodicity in the *x* direction, and periodicity in the *y* direction were fixed at *L*_*x*_ = 5.2 µm, *L*_*y*_ = 80 nm, *P*_*x*_ = 5.6 µm, and *P*_*y*_ = 0.8 µm, respectively. When *L*_*x*_ is fixed, the area enclosed by the arms, through which the magnetic field directly interacts with the bianisotropic SSRRs and DSRRs, will not significantly change after varying the pad shift (*X*). The pad dimensions determine the difference between the SSRR and DSRR. In the SSRR, the pad width and height are *W* = 1 µm and *H* = 0.4 µm, respectively. In the DSRR, the pad is split into two square pads with a side length of 0.4 µm separated by a 0.2 µm gap (*G*). We represent the symmetric SSRR and DSRR samples (with equal arm lengths of 2.4 µm) by S1 and D1, respectively. The asymmetric samples S2(D2) and S3(D3) are obtained by setting the pad shift to *X*_1_ = 0.8 µm and *X*_2_ = 1.6 µm, respectively. Following Ni/Au deposition and isotropic etching, the planner arms are released and bent under the residual stress to form vertical resonators. The top-right and bottom inset of Figure 1A–F show 60° and 85° (inset) side-view scanning electron microscopy (SEM) images of the self-folded SSRRs and DSRRs. The metal-stress self-folding method and approach for controlling the bending condition are detailed in the Methods section and Figure S1 of the Supporting Information.

### 2.2 Observation of scattered waves and radiation patterns of the symmetric and asymmetric vertical metamaterials

**Figure 2:**
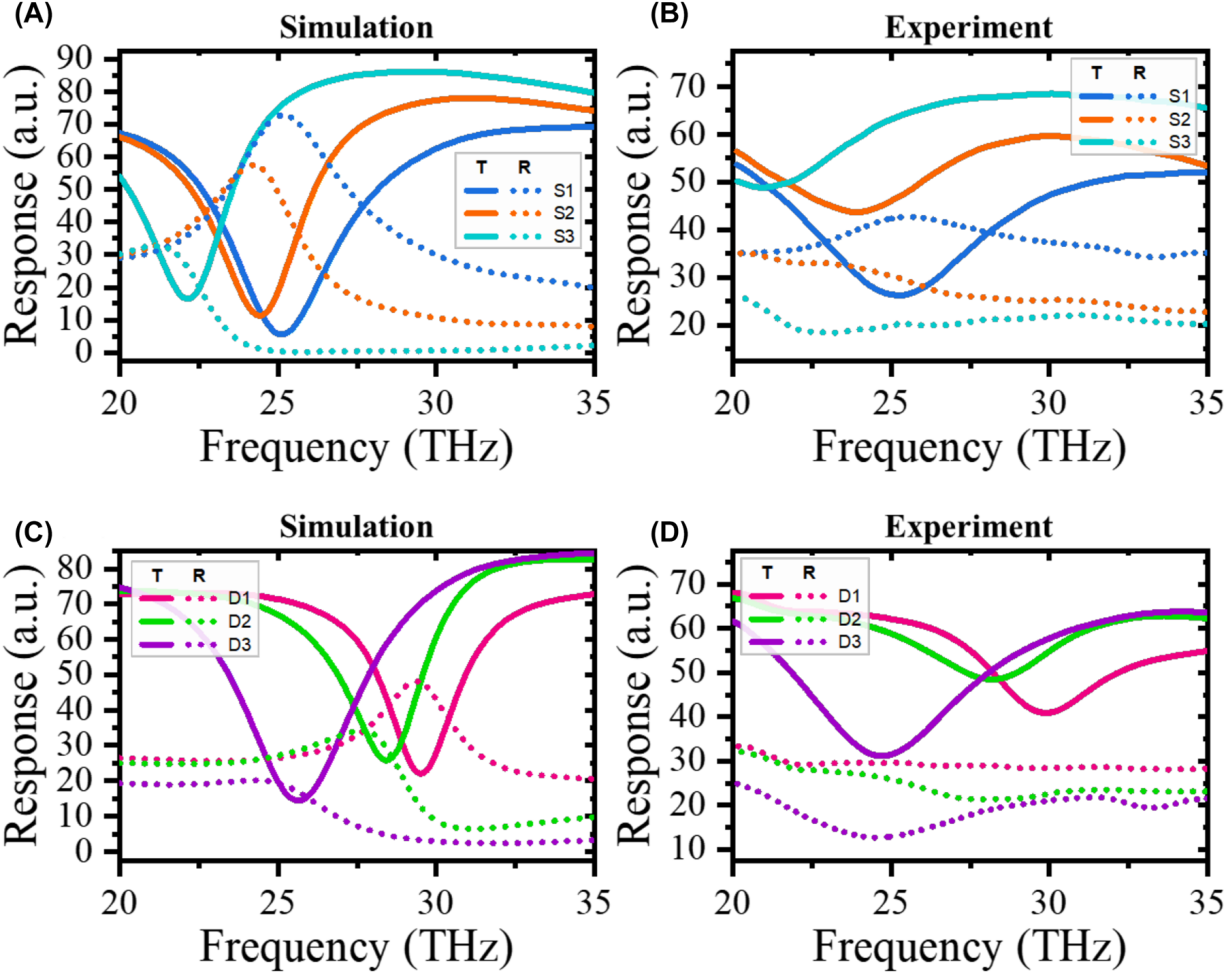
Photoresponse spectra of the SSRRs. (A) Simulated and (B) experimental transmittance (solid lines) and reflectance (dashed lines) spectra of the SSRRs (S1–S3) and (C and D) DSRRs (D1–D3).

The far-field radiation patterns are controlled by spatially varying the split direction of the SRRs. To this end, we change the ratio of the arm lengths on each side. Figure 2 shows the simulated and experimental transmittance and reflectance spectra of the SSRRs and DSRRs under normally incident light polarized along the lengthwise direction of the split. The numerical simulation was performed in COMSOL Multiphysics, which solves the 3D Maxwell equations using the finite-element approach. To completely analyze the scattering, radiation, transmittance, and reflectance properties of the metamaterials, the simulations were set up in a scattered field and a full field. The simulated geometries of the SSRRs and DSRRs were based on the 5°-, 65°-, and 85°-tilted SEM images of the fabricated structures (Figures 1C and F and S2). The refractive indices of the evaporated Au resonators and the crystalline Si substrate were set to their experimentally determined values [28]. The simulation setup is detailed in the Methods section. The transmittance and reflectance spectra were experimentally measured using Fourier transform infrared (FTIR) spectroscopy. After normalizing the raw spectra of the samples to those of air and 200 nm-thick Au film, the transmittance and reflectance were ∼70 % and 30 %, respectively, in the non-resonance region owing to the refractive index of the substrate, as dictated by the Fresnel equations.

In the simulation results of the SSRRs (i.e., S1–S3), the resonance strengths (indicated by the transmittance dips and reflectance peaks) and the resonance-frequency red shifts decreased monotonically with pad shift from S1 to S3 condition (Figure 2A). As the pad shift increases, the floating split rotates as indicated in Figure 1A. The tilted floating split shortens the effective gap, increasing the capacitance and thereby reducing the interaction with the electric field and the redshift. Note that the capacitance is related to the resonant frequency as $\omega \propto 1/\sqrt{\text{LC}}$ . The measurement results are shown in Figure 2B. In both the simulated and experimental results, the resonance strength and frequency show similar monotonic trends. The simulated resonance frequency and reflectance values also decrease monotonically from D1 to D3 (Figure 2C), but the transmittance dips increase from D1 to D2 and decrease from D2 to D3. The experimental results (Figure 2D) are consistent with the simulated results. In addition, the full-field simulation results well agree with the experimental transmittance and reflectance data.

**Figure 3:**
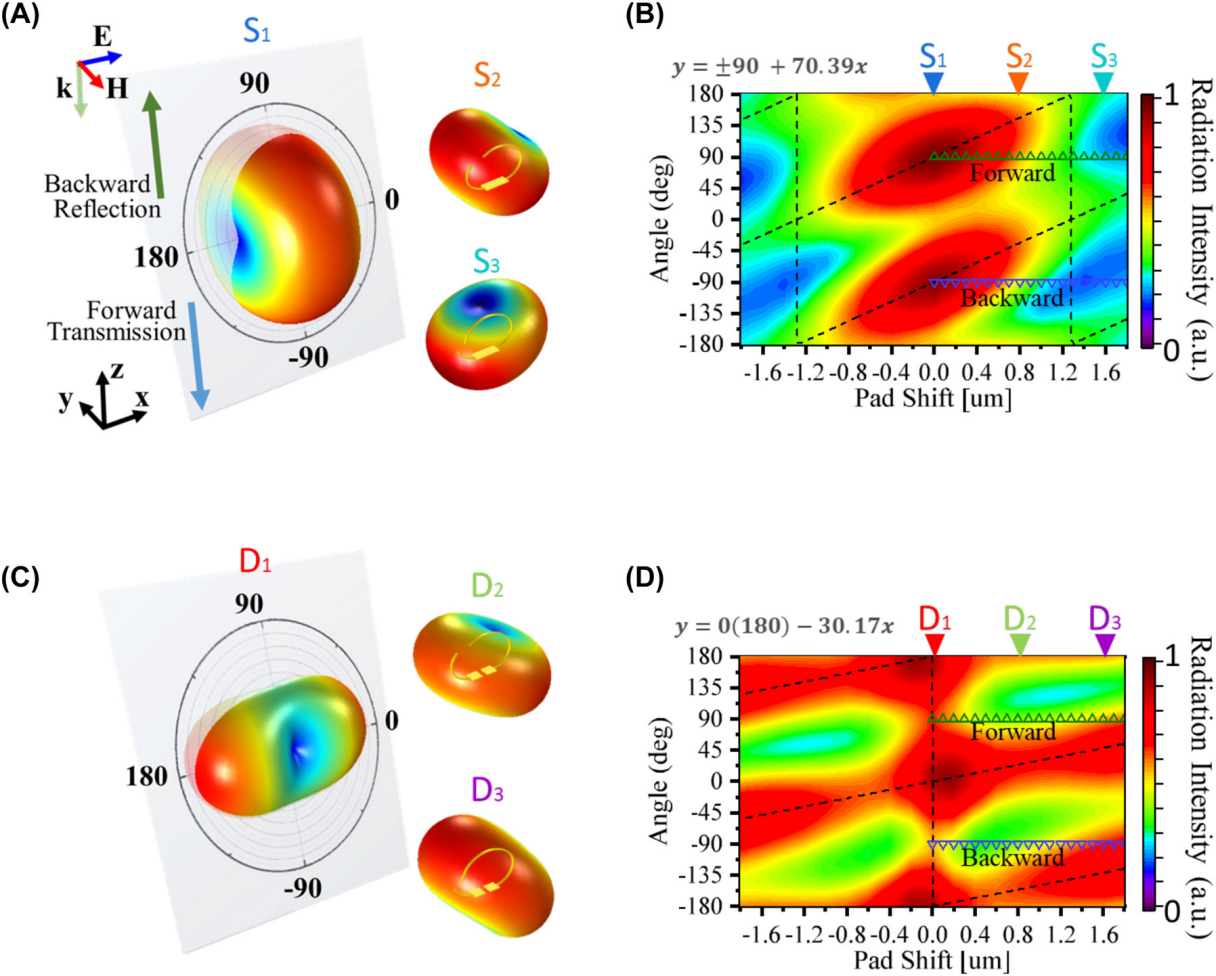
Far-field radiation powers of SSRRs and the intensities versus pad shift. (A) Maximum 3D far-field radiation powers of SSRRs with 0, 0.8 and 1.6 μm pad shifts. The *x*–*z* cross-sectional plane indicated the *E* plane radiation pattern. The backward and forward directions are indicated as 90° and −90° in the cross-sectional plane, respectively. (B) Radiation field intensities of the SSRRs versus pad shift, mapped in the *x*–*z* plane to illustrate their angular reconfigurations. The black dotted lines show the relation between the local radiation power maximum and the pad shift and the relation can be fitted as a linear relation shown on the figure. (C) and (D) As for (A) and (B), respectively, but for the DSRRs. The blue and green hollow triangles indicate the forward and backward directions, respectively.

Next, we explored light scattering from the vertical metamaterials and whether the metamaterials can reconfigure the radiation pattern. For this purpose, we employed a scattered field. Figure 3A displays the simulated 3D far-field radiation patterns of the SSRRs with respect to pad shift. The donut-shaped patterns suggest that the SSRRs act as simple dipole antennae and that the radiation is primarily contributed by the ED. The radiation pattern of each pad shift was obtained at the resonant frequency, which maximizes the scattered radiation power. In the *x*–*z* cross-sectional plane of S1, the *E*-plane of the radiation pattern and the maximum radiation intensity appear in the −90° and 90° directions, indicating that S1 scattering propagates along the backward and forward directions. As shown in the radiation patterns of S1–S3 (Figure 3A), the donut-shaped radiation pattern rotates along the *y* axis with increasing pad shift. Section 2 of the Supporting Information show the *x*–*z* cross-sectional radiation pattern overlay of the SSRRs and DSRRs with different pad shift. The relation with angular, field intensity, and the pad shift shown in Figure S3A and B can be plot as Figure 3B and D. Figure 3B maps the radiation-field intensity in the *x*–*z*-plane with respect to pad shift. At pad shifts of 0.8 µm (S2) and 1.6 µm (S3), the radiation intensity is maximized at 130° and −160° (200°), respectively. When the radiation angles of maximum intensity were fitted as linear functions of the pad shift in the forward and backward scattering directions of the SRRS, the slope was obtained as 70.39°/μm, clearly suggesting that the scattering direction can be configured by tailoring the geometry of our vertical SRRs.

The 3D radiation patterns of the DSRRs are illustrated in Figure 3C. In contrast to S1, D1 radiates along the 0° and 180° directions. Moreover, the radiation pattern of D1 differs from the donut-shaped radiation pattern of D2 and D3, implying the induction of multipoles in D1 and dipoles in D2 and D3. In the mapped *x*–*z* plane of the DSRRs (Figure 3D), the radiation intensities of D2 and D3 are maximized at 152° and 136°, respectively, and the plot of angle versus pad-shift of the DSRRs has a gradient of 30.17°/μm. Although the angle is less sensitive to pad shift in the DSSRs than in the SSRRs, the DSRRs can scatter the normal incident wave into the plane direction under the geometrically symmetric condition (D1). Overlaying the SSRRs and DSRRs provides a comprehensive angular controllability in which the pad-shift region can be lowered to ±0.9 μm, thus allowing whole angle (0–360°) radiation reconfiguration (see Figure S3C). However, experimentally proving this phenomenon is difficult because the setup cannot detect the angle-resolved scattering pattern in the mid-IR region.

**Figure 4:**
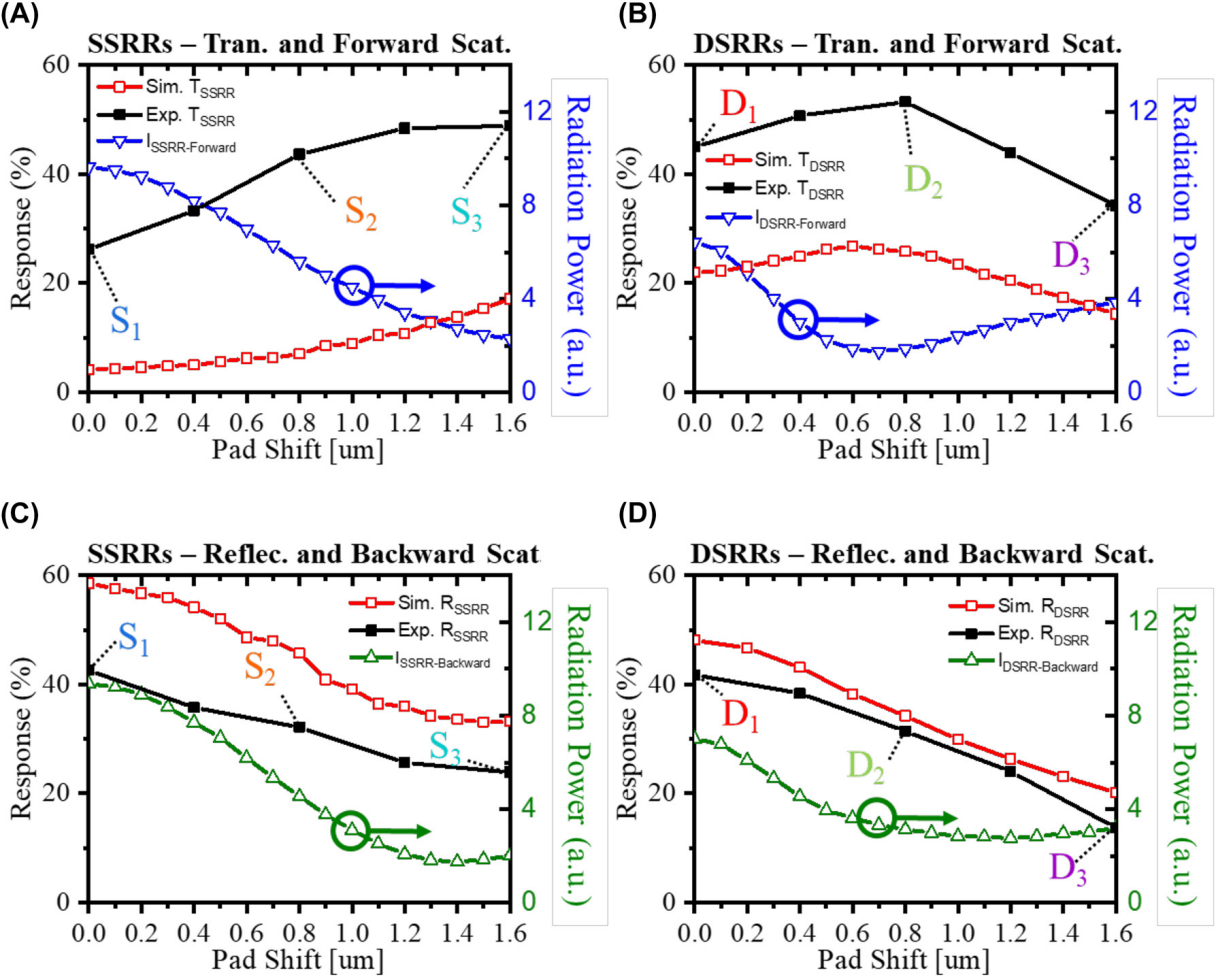
Relation between transmittance and far-field radiation powers along forward direction under resonance frequency of (A) SSRRs and (B) DSRRs; reflectance and far-field radiation powers backward direction of (C) SSRRs and (D) DSRRs.

The responses of transmittance and reflectance under resonance frequency with different pad shifts in Figure 2 and the radiation powers at ±90° as forward and backward directions (blue and green triangles in Figure 3B and D were extracted and shown in Figure 4. The purpose is to compare the full field simulation results (transmission and reflection) with the scattered field ones (interaction between metamaterials and incident light) and also connected with the experimental transmittance and reflectance. The simulated transmitance trends of the SSRRs and DSRRs match the experimental results and are inversely related to the forward-scattered (90°) radiation powers (Figure 4A and B. Meanwhile, the ones extracted from the reflectance results and the backward-scattered radiation power at −90° (Figure 4C and D) all decrease with increasing pad shift. The inverse relation between forward and backward radiation power and the correlated transmittance and reflectance trend can be explained by destructive interference between the light scattered by the metamaterials and the light transmitted by the substrate in the full field. Therefore, strong resonance and light scattering from the resonators lead to low transmittance dips. On the contrary, as less light is reflected from the substrate than is transmitted through the substrate, the reflectance is enhanced by backward scattering of the stronger metamaterial. To observe the angular reconfiguration with the symmetric and asymmetric SSRRs and DSRRs, a grazing incidence reflection module is utilized to characterize the angular signal, as shown in Figure S7. The measured twice oblique incidence and reflection results show that only the symmetric SSRRs and DSRRs could have reflection signals. The cartoon figures in Figure S7B, C, D, and F connected the radiation pattern of S1(D1) and S3(D3) with the incidence angle which explained the reflection intensity difference is due to the symmetric and asymmetric radiation patterns. Despite that there is a lack of metrology to comprehensively analyze scattered radiation patterns in mid-infrared, with the relationship between the scattered-field radiation patterns and responses (transmittance, reflectance, and grazing incidence reflection) of the resonators in the full-field simulation and experiments, we can connect the transmitance/reflectance response with the forward and backward radiation power change which partially proved the angular reconfiguration by tailoring the geometrical parameters.

### 2.3 Unveiling the origin of angular reconfiguration metamaterials through multipole expansion

The origin of the angular reconfiguration was elucidated using the multipole expansion method, in which the resonator’s current density is spatially integrated into the scattering-field mode of the simulation to retrieve the multipole moments and radiation power. Section 3 of the Supporting Information describes the extraction of the multipole-moment power and Figure S4 displays the field distributions and current densities of the SSRRs and DSRRs. The total radiation power contributed by each multipole moment is given by [27]:

(2) $$\begin{array}{ll} I & =\frac{2\omega^{4}}{3c^{3}}{\left|\vec{P}\right|}^{2}+\frac{2\omega^{4}}{3c^{3}}{\left|\vec{M}\right|}^{2}+\frac{4\omega^{5}}{3c^{4}}\left(\vec{P}\cdot \vec{T}\right)+\frac{2\omega^{6}}{3c^{5}}{\left|\vec{T}\right|}^{2}\\ & \;+\frac{\omega^{6}}{5c^{5}}\sum {\left|\vec{Q_{\alpha \beta }}\right|}^{2}+\frac{\omega^{6}}{40c^{5}}\sum {\left|\vec{M_{\alpha \beta }}\right|}^{2} \end{array}$$

where *P*, *M*, *T*, *Q*, and *M* denote the ED, MD, toroidal dipole (TD), EQ, and magnetic quadrupole moment, respectively, *ω* represents the angular frequency, *c* represents the speed of light in vacuum, and *α* and *β* represent the corresponding coordinates (*x*, *y*, *z*).

**Figure 5:**
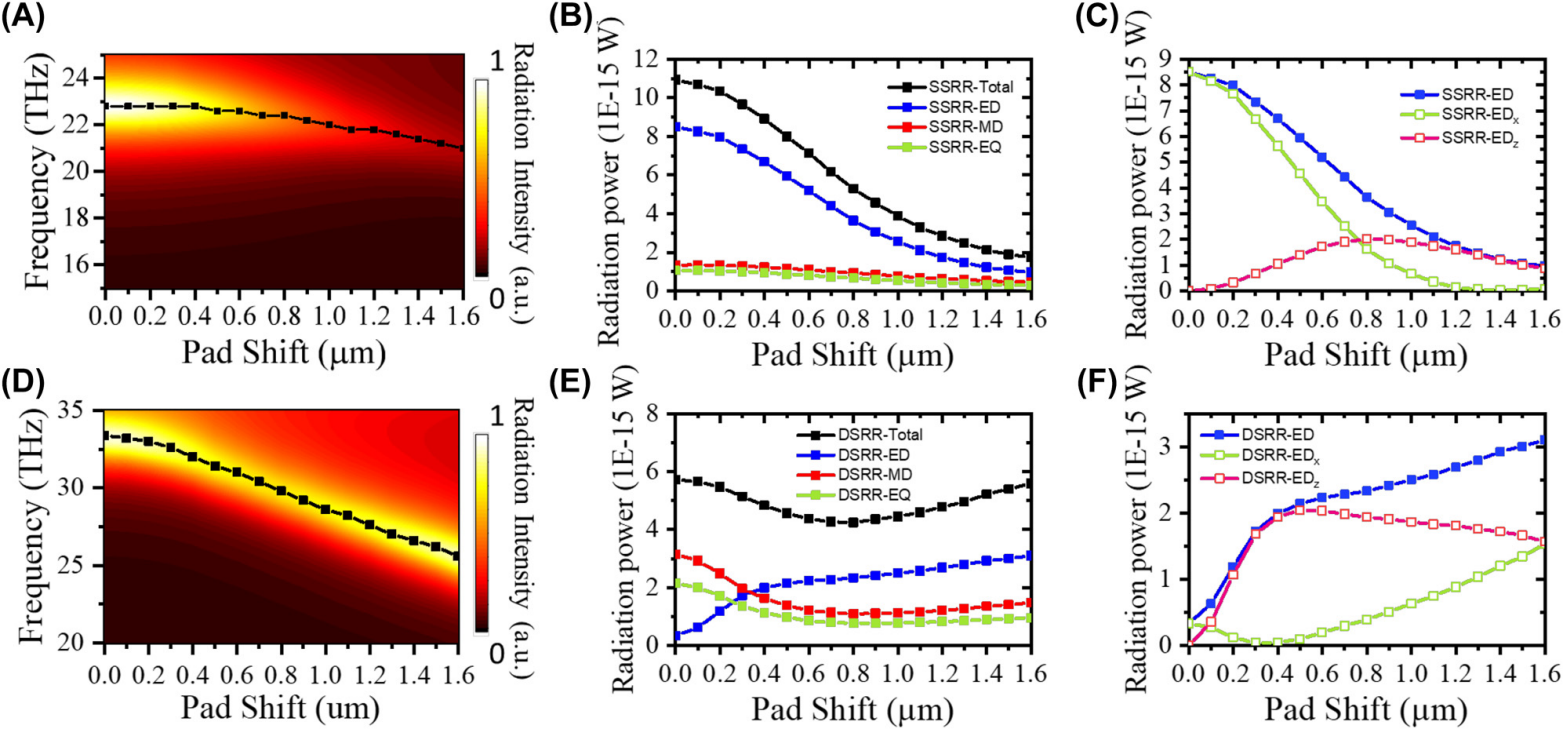
Radiation-power intensity and spectra using the multipole expansion method. (A) Mapped total radiation-power intensity of SSRRs, (B) contributions of the electric dipole (ED, blue), magnetic dipole (MD, red), and electric quadrupole (EQ, green) to the total radiation power (black); (C) the ED (blue) can be separated into *x* and *z* components (ED_*x*_ (green) and ED_*z*_ (red), respectively). (D)–(F) As for (A)–(C), but for the DSRRs. In (A) and (D), bright coloration indicates a high total radiation power and the black dots plot the frequency at which the radiation power is maximized at each pad shift.

Figure 5A plots the total-radiation power spectrum of the SSRRs normalized to the maximum radiation power in the symmetric SSRRs. To investigate the intensity decline and redshift with increasing pad shift, the contributions of different poles were extracted from the maximum total radiation power. The results are presented in Figure 5B. The total radiation power is dominated by ED radiation under both symmetric and asymmetric conditions, which explains the origin of the donut-shaped radiation pattern in Figure 3A.

To examine the angular reconfiguration, the ED radiation power was decomposed into its ED_*x*_ and ED_*z*_ components acting along the *x* and *y* directions, respectively (Figure 5C). Under the symmetric condition, the floating split of the SSRR is directed along the polarization of the background plane wave, resulting in strong ED_*x*_ radiation. As the pad shift increases, the floating split rotates around the center of the ring resonator. The strong bianisotropic vertical SSRRs directly interact with both the incident electric and magnetic fields, inducing the ED along the rotated floating split. Consequently, the ED_*z*_ strengthens with increasing pad shift while the ED_*x*_ weakens. The ED_*z*_ slightly exceeds the ED_*x*_ at a pad shift of 0.8 µm, which explains why the 3D radiation pattern of S2 (Figure 3A) is directed along 130° (near 135°). When the pad shift exceeds 1.2 µm, the ED_*x*_ vanishes and ED_*z*_ dominates the scattering; accordingly, the ED tilts by 90° and radiates along the in-plane direction.

## 3 Conclusions

In this study, we achieved angular reconfiguration by tailoring the vertical geometries in the mid-IR region with two types of vertical metamaterial structures, SSRRs and DSRRs. Vertical metamaterials can be efficiently fabricated using the metal-stress-driven self-folding method, which can design an asymmetric vertical structure using different planar pattern designs. By designing unequal arm lengths, we can tailor the angle of the floating split of the SSRRs and DSRRs and control the compositions of the electromagnetic multipoles. As the pad shift increased, the measured transmittances and reflectances of the SSRRs and DSRRs exhibited the same trends as the simulated ones. From the simulated far-field radiation patterns, we determined that the SSRRs and DSRRs can reconfigure scattered light propagation from 90° to 212° and −32° and from 0° to 62° and −56°, respectively. Furthermore, by decomposing the multipoles into their constituents, we found that the directional scattering performance can be verified by manipulating the electric dipoles in the *x* and *z* directions (i.e., the ED_*x*_ and ED_*z*_ components). The combination of SSRRs and DSRRs thus offers an effective, efficient, and unique approach for reconfiguring the radiation direction in the IR region, enabling its application in compact optical metadevices.

## 4 Methods

### 4.1 Sample preparation

The vertical metamaterials were fabricated as follows. The electron beam resist (polymethyl methacrylate A4) was spin-coated on a Si substrate, cleaned with acetone and isopropanol (IPA) for 10 min, centrifuged at 4000 rpm for 40 s, and baked at 160 °C for 10 min on a hotplate. The 2D patterns were then defined using an electron beam lithography system (Elionix ELS-7500EX, province, country) with an accelerating voltage of 100 kV, a beam current of 100 pA, a field size of 600/60,000 μm/dot, and an exposure dosage of 3.4 μs/dot. The exposed resist was developed in a mixture of methyl isobutyl ketone and IPA (1:3) for 75 s followed by IPA for 25 s. Ni/Au (5/50 nm) films were then deposited using a thermal evaporator with a coating rate of 0.3/1.0 Å/s under a pressure of 3 × 10^−6^ Torr. The lift-off process was conducted by dissolving the resist on the substrate in acetone. The vertical metamaterials in the substrate were created by isotropically etching the bottom Si substrate and the arms were released in an inductively coupled plasma (ICP) reactive-ion etching system (Plasmalab System 100, Oxford) with a C_4_F_8_ plasma flow rate of 25 sccm, a pressure of 0.15 Pa, an ICP power of 2000 W, and a forward power of 0 W for 270 s.

### 4.2 Optical response characterization

The transmittance and reflectance spectra of the fabricated structures were measured with an FTIR spectrometer (Bruker VERTEX 70v) equipped with a microscope (magnification ×15) and an objective lens with a numerical aperture of 0.4. The measurement was performed in the mid-IR region (400–4000 cm^−1^) under linearly polarized incident light at a normal incidence angle. The HgCdTe detector was cooled with liquid nitrogen to enhance the sensitivity. The measurement area was (100 × 100) μm^2^ with 256 integration times and an 8 cm^−1^ frequency resolution. The transmittance and reflectance spectra were normalized to the spectrum in air and to the total reflection spectrum of the sample (200 nm-thick Au film on the Si substrate), respectively. A grazing angle objective is utilized to observe the oblique incidence and reflectance. The objective with polarizer allows the light to pass through the sample twice with p-polarized incidence and reflectance to ∼84°.

### 4.3 Simulations

During the simulations, Maxwell’s equations were numerically solved using the finite-element approach in COMSOL Multiphysics. To obtain sufficient information for demonstrating the angular reconfiguration under SSRR and DSRR conditions, we executed both full-field and scattered-field simulations. The full-field simulation setup was intended to theoretically confirm the measured transmittance and reflectance spectra. The refractive index of Si is 3.5 and that of Au was obtained from Olmon et al. [28] who investigated evaporated Au thin films in the IR region. Along the *x* and *y* axes, we imposed Floquet periodic boundaries with periodicities of 5.6 and 0.8 µm, respectively. Along the *z-*axis, the boundary conditions were periodic ports with normally incident linearly polarized illumination along the split direction of the symmetric SSRRs and DSRRs. The two periodic ports were separated by 30 µm and the vertical metamaterial was placed in the middle of the domain. The dimensions and shapes of the vertical SSRRs and DSRRs were based on the SEM images taken at stage-tilted angles of 5°, 60°, and 85°. The height of the remaining Si pillar beneath the pad of the ring resonator was set to 0.4 µm. The metamaterials and ports were separated by 15 µm, longer than the excitation wavelength. The radiation patterns are extracted based on scattered field simulation. The scattered-field simulation was set up with the same geometries as the full-field simulation. The primary scattering domain was covered with a perfectly matched 1 µm-thick layer to absorb the light approaching the boundary and to accurately describe the interaction between the linearly polarized light and metamaterials. Note that a far-field domain was constructed to extract the far-field radiation pattern.

## Supporting Information

Fabrication and curvature optimization via the metal-stress self-folding method; tailoring the vertical metamaterials geometries to achieve angular reconfiguration; multipole expansion using the numerical current density.

## Supplementary Material

Supplementary Material Details

## References

[1] Yen T. J., Padilla W. J., Fang N. (2004). Terahertz magnetic response from artificial materials. *Science*.

[2] Valentine J., Zhang S., Zentgraf T. (2008). Three-dimensional optical metamaterial with a negative refractive index. *Nature*.

[3] Pitchappa P., Kumar A., Liang H. (2020). Frequency‐agile temporal terahertz metamaterials. *Adv. Opt. Mater.*.

[4] Poulikakos L. V., Thureja P., Stollmann A., De Leo E., Norris D. J. (2018). Chiral light design and detection inspired by optical antenna theory. *Nano Lett.*.

[5] Li N., Lai Y., Lam S. H., Bai H., Shao L., Wang J. (2020). Directional control of light with nanoantennas. *Adv. Opt. Mater.*.

[6] Baryshnikova K. V., Smirnova D. A., Luk’yanchuk B. S., Kivshar Y. S. (2019). Optical anapoles: concepts and applications. *Adv. Opt. Mater.*.

[7] Cui C., Yuan S., Qiu X. (2019). Light emission driven by magnetic and electric toroidal dipole resonances in a silicon metasurface. *Nanoscale*.

[8] Ginel-Moreno P., Sanchez-Postigo A., de-Oliva-Rubio J. (2021). Millimeter-long metamaterial surface-emitting antenna in the silicon photonics platform. *Opt. Lett.*.

[9] Martins T., Cui Y., Gholipour B., Ou J. Y., Frazão O., MacDonald K. F. (2021). Fiber‐integrated phase change metasurfaces with switchable group delay dispersion. *Adv. Opt. Mater.*.

[10] Vercruysse D., Neutens P., Lagae L., Verellen N., Van Dorpe P. (2017). Single asymmetric plasmonic antenna as a directional coupler to a dielectric waveguide. *ACS Photonics*.

[11] Lazarides N., Tsironis G. P. (2018). Superconducting metamaterials. *Phys. Rep.*.

[12] Alaee R., Albooyeh M., Tretyakov S., Rockstuhl C. (2016). Phase-change material-based nanoantennas with tunable radiation patterns. *Opt. Lett.*.

[13] Luo Y., Chu C. H., Vyas S. (2021). Varifocal metalens for optical sectioning fluorescence microscopy. *Nano Lett.*.

[14] Ren Z., Chang Y., Ma Y., Shih K., Dong B., Lee C. (2019). Leveraging of MEMS technologies for optical metamaterials applications. *Adv. Opt. Mater.*.

[15] Tanaka T., Yano T.-a., Kato R. (2022). Nanostructure-enhanced infrared spectroscopy. *Nanophotonics*.

[16] Tanaka Y. Y., Shimura T. (2017). Tridirectional polarization routing of light by a single triangular plasmonic nanoparticle. *Nano Lett.*.

[17] Vaskin A., Bohn J., Chong K. E. (2018). Directional and spectral shaping of light emission with mie-resonant silicon nanoantenna arrays. *ACS Photonics*.

[18] Dregely D., Taubert R., Dorfmuller J., Vogelgesang R., Kern K., Giessen H. (2011). 3D optical Yagi-Uda nanoantenna array. *Nat. Commun.*.

[19] Alaee R., Filter R., Lehr D., Lederer F., Rockstuhl C. (2015). A generalized Kerker condition for highly directive nanoantennas. *Opt. Lett.*.

[20] King N. S., Knight M. W., Large N., Goodman A. M., Nordlander P., Halas N. J. (2013). Orienting nanoantennas in three dimensions to control light scattering across a dielectric interface. *Nano Lett.*.

[21] Liu Z., Cui A., Li J., Gu C. (2019). Folding 2D structures into 3D configurations at the micro/nanoscale: principles, techniques, and applications. *Adv. Mater.*.

[22] Tsai H.-Y., Chen C.-C., Chen T.-A., Tsai D. P., Tanaka T., Yen T.-J. (2020). Realization of negative permeability in vertical double split-ring resonators with normal incidence. *ACS Photonics*.

[23] Yoon G., Kim I., So S., Mun J., Kim M., Rho J. (2017). Fabrication of three-dimensional suspended, interlayered and hierarchical nanostructures by accuracy-improved electron beam lithography overlay. *Sci. Rep.*.

[24] Liu Z., Du S., Cui A. (2017). High‐quality‐factor mid‐infrared toroidal excitation in folded 3D metamaterials. *Adv. Mater.*.

[25] Jung W., Jung Y. H., Pikhitsa P. V. (2021). Three-dimensional nanoprinting via charged aerosol jets. *Nature*.

[26] Chen C.-C., Ishikawa A., Tang Y.-H., Shiao M.-H., Tsai D. P., Tanaka T. (2015). Uniaxial-isotropic metamaterials by three-dimensional split-ring resonators. *Adv. Opt. Mater.*.

[27] Radescu E. E., Vaman G. (2002). Exact calculation of the angular momentum loss, recoil force, and radiation intensity for an arbitrary source in terms of electric, magnetic, and toroid multipoles. *Phys. Rev. E: Stat., Nonlinear, Soft Matter Phys.*.

[28] Olmon R. L., Slovick B., Johnson T. W. (2012). Optical dielectric function of gold. *Phys. Rev. B*.

