# Supplementary material for: Manipulation on radiation angles via spatially organized multipoles with vertical split-ring resonators
Source: Nanophotonics. 2023 Oct 5;12(20):3921–30. doi: 10.1515/nanoph-2023-0386 (PMC11501352; doi:10.1515/nanoph-2023-0386)
Supplement: Supplementary file 1 — Supplementary Material Details [file j_nanoph-2023-0386_suppl_001.docx]

Hao-Yuan Tsai^1,2,3^, Che-Chin Chen^2,^*, Chun-Yen Chen^1^, Yi-Jie Lin^1^, Wei-Chun Chen^2^, Hung-Pin Chen^2^, Yu-Wei Lin^2^, Takuo Tanaka^3,4^, and Ta-Jen Yen^1,*^

Manipulation on radiation angle via spatially organized multipoles with vertical split-ring resonators

*Supporting Information*

1. Design and curvature optimization using the metal-stress self-folding method


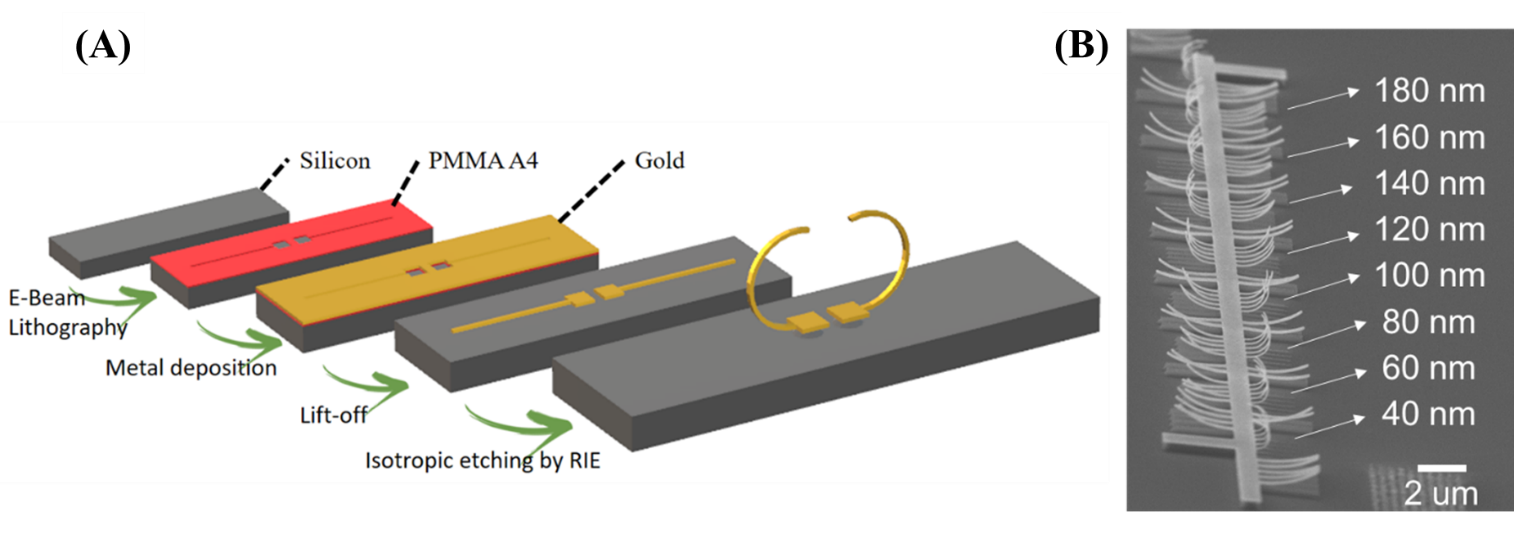


**Figure S1.** Schematic of the (A) fabrication process and (B) the test sample designs of Ni/Au (5/50 nm) with arm widths ranging from 40 to 180 nm.

**Figure S1A** depicts the detailed fabrication conditions. The specific curve of the cantilever can be designed with different arm widths and lengths using different etching techniques. **Figure S1B** shows the test sample with a fixed arm length (2 µm) and different widths (20–180 nm) for optimizing the isotropic etching recipe. The findings suggest that narrowing the arms of the structure increases the curvature of bending. **Figure S2** shows the 5°-tilted SEM images of the fabricated SSRRs and DSRRs.


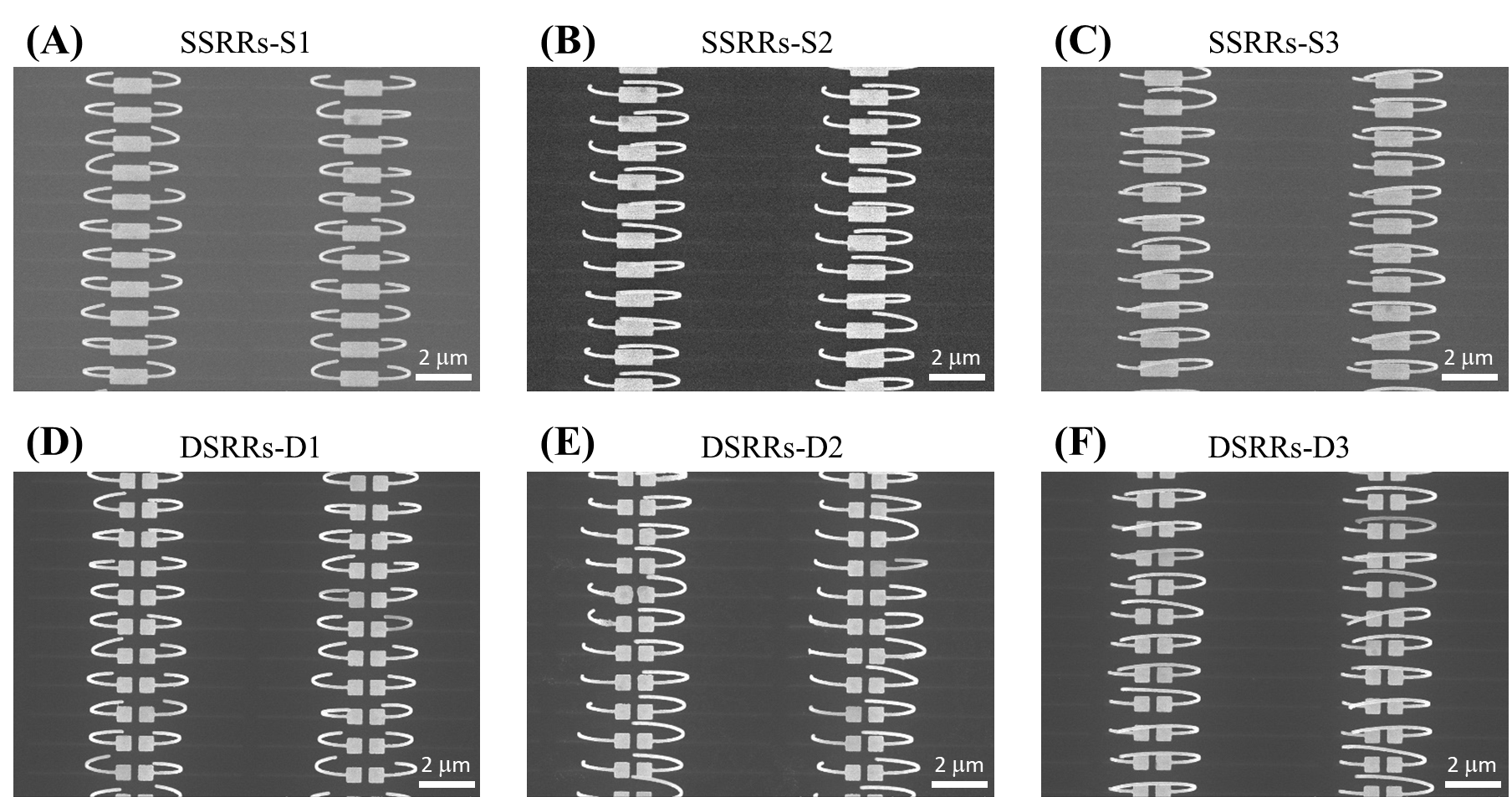


**Figure S2.** 5°-tilted SEM images of the designed SSRRs: (A) S1, (B) S2, and (C) S3 and the designed DSRRs: (D) D1, (E) D2, (F) D3.

1. Achieving angular reconfiguration by modifying the morphologies of the vertical metamaterials


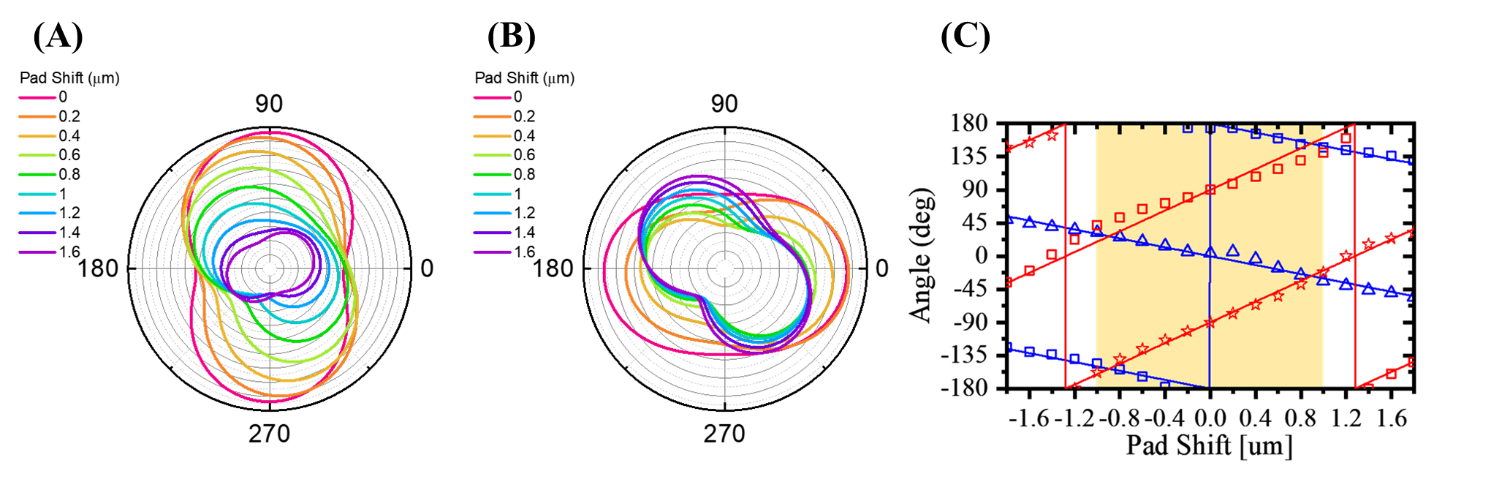


**Figure S3.** 2D radiation patterns in the *x–y* plane of (A) SSRRs and (B) DSRRs with different pad shifts. (C) In Figure 3(B) and (D) of the main text, the fitted lines of the SSRRs and DSRRs overlap the maximum radiation power. The yellow region indicates the minimum pad-shift region in which the configuration changes from 0 ° to ±180 ° by combining the SSRRs and DSRRs.

1. Multipole Expansion using the Numerical Current Density

To find the multipole moment and radiation power, we integrated the simulated current density as follows:

The electric dipole moment and its radiation power are respectively given by

$\vec{P}=\frac{1}{i\omega}\int d^{3}\vec{j}$, $I_{p}=\frac{2\omega^{4}}{3c^{3}}\left| \vec{P} \right|^{2}$ . (S1)

The magnetic dipole moment and its radiation power are respectively given by

$\vec{M}=\frac{1}{2c}\int d^{3}r\left( \vec{r}\times\vec{j} \right)$, $I_{m}=\frac{{2\omega}^{4}}{{3c}^{3}}\left| \vec{M} \right|^{2}$ . (S2)

The toroidal dipole moment and its radiation power are respectively given by

$\vec{T}=\frac{1}{2c}\int d^{3}r\left[ \left( \vec{r}\cdot\vec{j} \right)r-2r^{2}\vec{j} \right]$, $I_{T}=\frac{{2\omega}^{6}}{{3c}^{5}}\left| \vec{T} \right|^{2}$. (S3)

The electric quadrupole moment and its radiation power are respectively given by

$\vec{Q_{\alpha\beta}}=\frac{1}{i2\omega}\int d^{3}r\left[ r_{\alpha}j_{\beta}+r_{\beta}j_{\alpha}-\frac{2}{3}\delta_{\alpha\beta}(\vec{r\cdot}\vec{j}) \right]$, $I_{Q}=\frac{\omega^{6}}{{5c}^{5}}\sum\left| \vec{Q_{\alpha\beta}} \right|^{2}$ . (S4)

The magnetic quadrupole moment and its radiation power are respectively given by

$\vec{M_{\alpha\beta}}=\frac{1}{3c}\int d^{3}r\left[ \left( \vec{r}\times\vec{j} \right)_{\alpha}r_{\beta}+\left( \vec{r}\times\vec{j} \right)_{\beta}r_{\alpha} \right]$, $I_{M}=\frac{\omega^{6}}{{40c}^{5}}\sum\left| \vec{M_{\alpha\beta}} \right|^{2}$ . (S5)

In these expressions, *ω* and *c* represent the angular frequency and speed of light, respectively, ***j*** denotes the current density at point (*x*, *y*, *z*), **r** denotes the distance vector from the origin to (*x*, *y*, *z*) in the Cartesian coordinate system, and $\alpha$ and $\beta$ represent the plane in which the quadrupole exists and is calculated.

The multipole radiation powers of the SSRRs and DSRRs with different pad shifts, extracted using the above-described method, are shown in **Figures S4 and S5**, respectively. In each figure, the line indicates the frequency at which the radiation power is maximized as a function of pad shift. The radiation power of each multipole in **Figure 5 (B, C, E, F)** was extracted from the value of the symbols in **Figures S4** and **S4**.


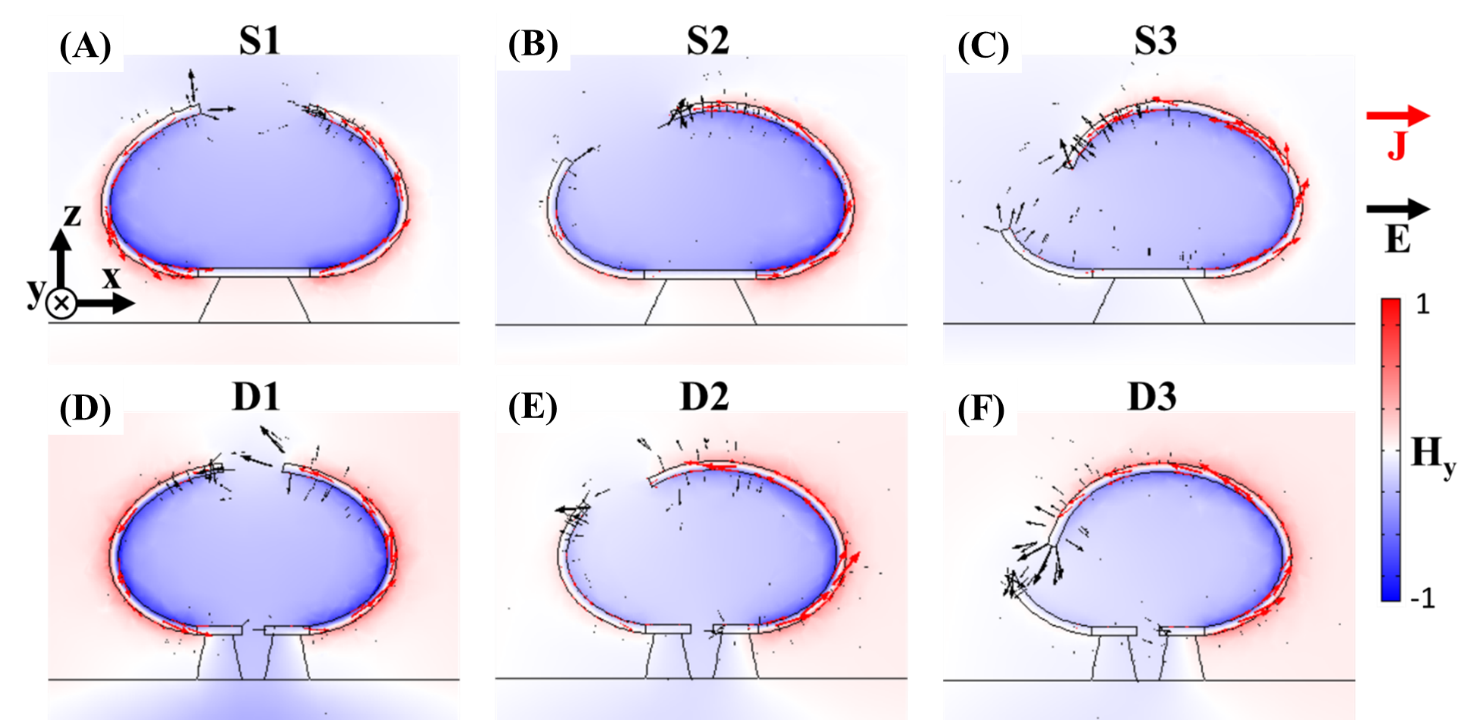


**Figure S4.** Normalized *y*-component of the magnetic field (*H_y_*), current density vectors, and electric field vectors of (A–C) SSRR and (D–F) DSRR


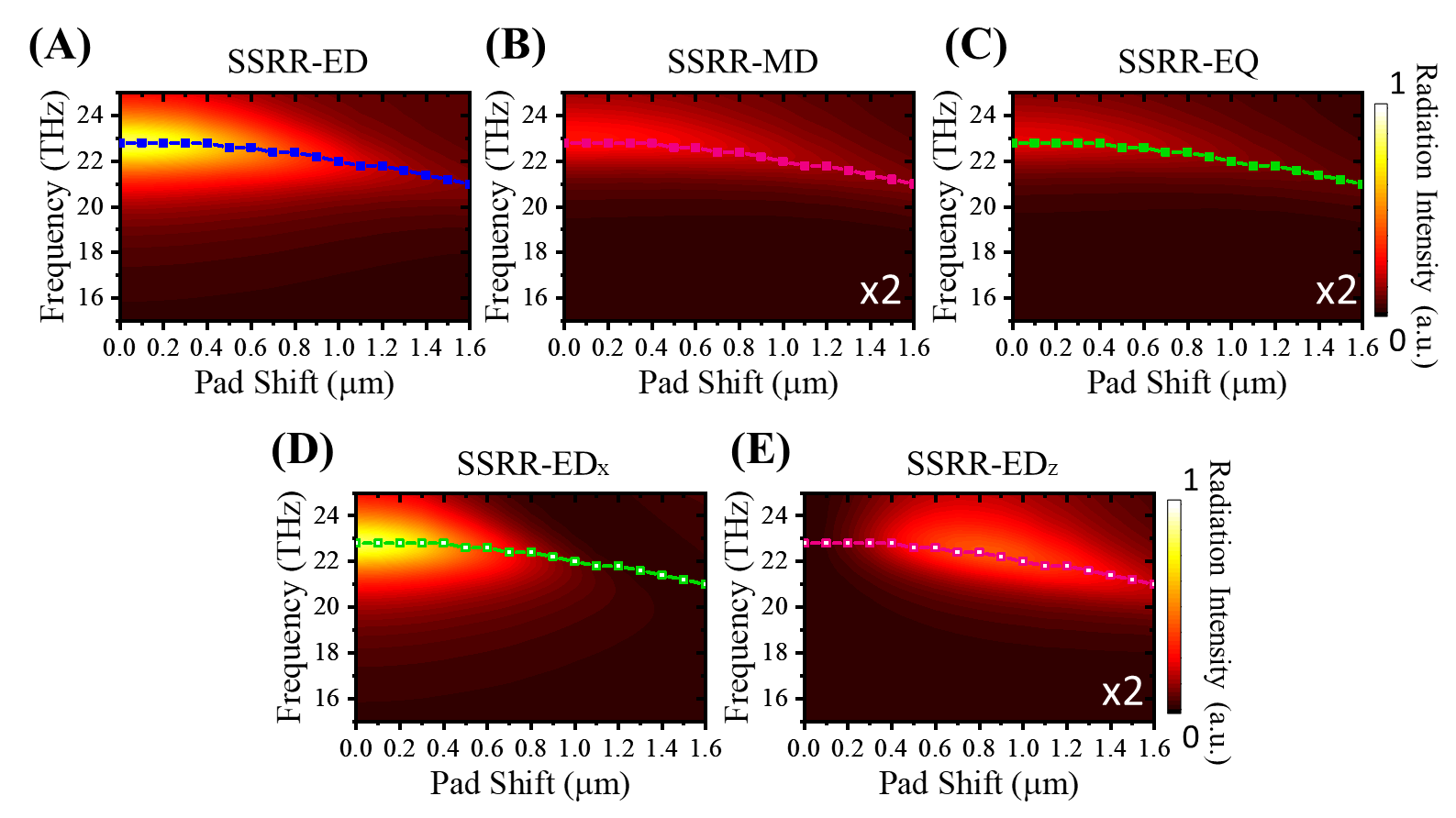


**Figure S5.** Radiation powers of (A) electric dipole, (B) magnetic dipole, and (C) electric quadrupole of the SSRR; (D) *x*-components (ED*_x_*) and (e) *z*-components (ED*_z_*) extracted from the electric dipole


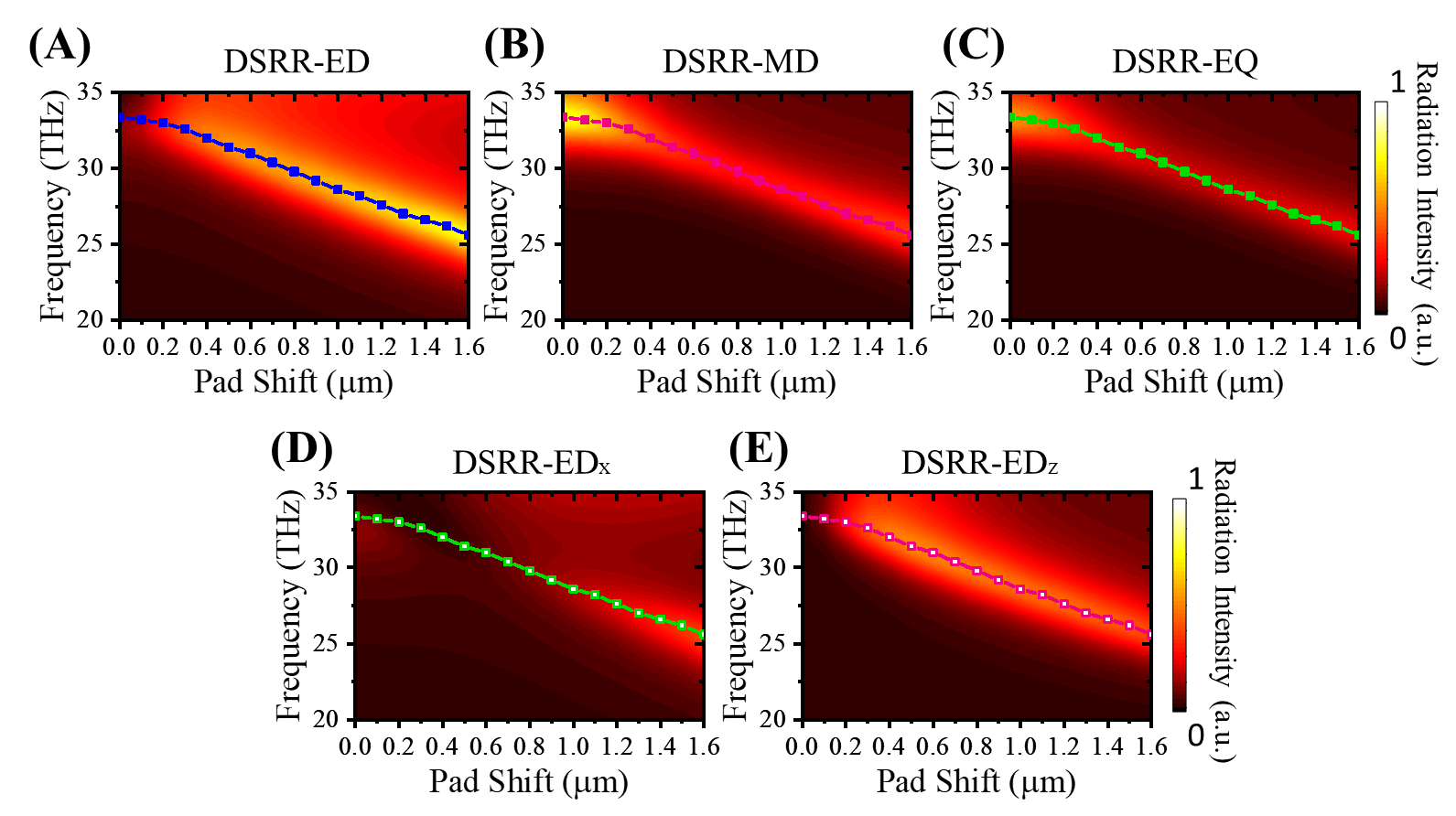


**Figure S6.** Radiation powers of (A) electric dipole, (B) magnetic dipole, and (C) electric quadrupole of the DSRR; (D) *x*-components (ED*x*) and (E) *z*-components (ED*_z_*) extracted from the electric dipole

**
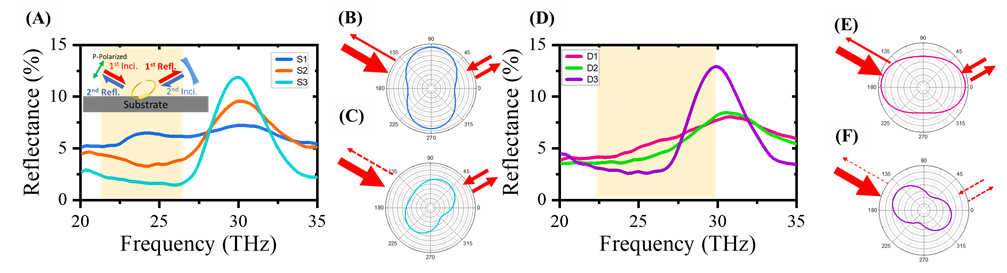
Figure S7**. Grazing incidence reflection for **(A)** SSRR and **(D)**DSRR with 0.4, 0.8, 1.2, 1.6, 2.0 um pad shift. The inset in **(A)** illustrates the grazing incident reflection module, which collects scattering signals under reflection twice, as shown in the inset figure. The cartoon figures of twice reflection response of symmetric and asymmetric SSRR are shown in **(B)** and **(C)**. The ones in DSRR are shown in **(E)** and **(F)**. The strong reflected peaks around 30 THz in both SSRRs and DSRRs are based on the LSPR mode of the periodic metamaterials due to the p-polarized light rather than the response of the meta-atom itself.
